# The Future of Influenza Vaccines: A Historical and Clinical Perspective

**DOI:** 10.3390/vaccines6030058

**Published:** 2018-08-30

**Authors:** Nicole M. Bouvier

**Affiliations:** 1 Division of Infectious Diseases, Department of Medicine, Icahn School of Medicine at Mount Sinai, New York, NY 10029, USA; nicole.bouvier@mssm.edu; Tel.: +1-212-241-1956; 2 Department of Microbiology, Icahn School of Medicine at Mount Sinai, New York, NY 10029, USA

**Keywords:** influenza, vaccine, transmission, morbidity, mortality, efficacy, effectiveness

## Abstract

For centuries, the development of vaccines to prevent infectious disease was an empirical process. From smallpox variolation in Song dynasty China, through the polysaccharide capsule vaccines developed in the 1970s, vaccines were made either from the pathogen itself, treated in some way to render it attenuated or non-infectious, or from a closely related non-pathogenic strain. In recent decades, new scientific knowledge and technologies have enabled rational vaccine design in a way that was unimaginable before. However, vaccines optimal against some infectious diseases, influenza among them, have remained elusive. This review will highlight the challenges that influenza viruses pose for rational vaccine design. In particular, it will consider the clinically beneficial endpoints, beyond complete sterilizing immunity, that have been achieved with vaccines against other infectious diseases, as well as the barriers to achieving similar success against influenza.

## 1. Introduction

**Figure 1:**
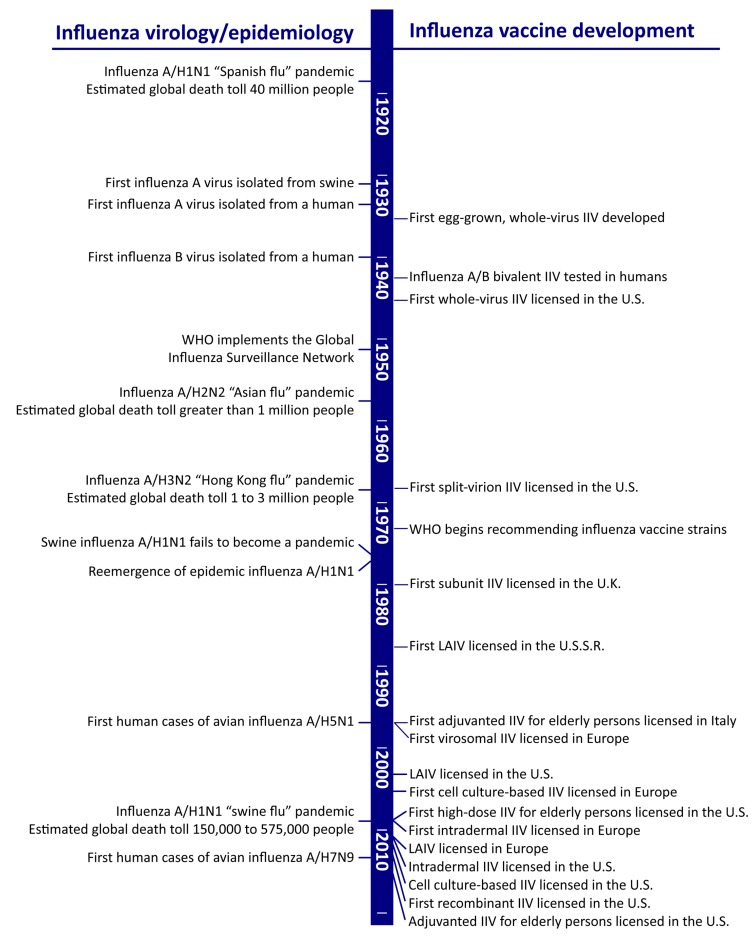
Milestones in the history of influenza vaccines [14,15,16,17,18,19,20,21,22,23,24,25]. IIV, inactivated influenza vaccine; WHO, World Health Organization; LAIV, live attenuated influenza vaccine.

The majority of currently licensed influenza vaccines are inactivated influenza vaccines (IIVs), based on purified viral protein components that are administered by intramuscular injection. The protein production and purification technologies vary according to manufacturer, but the general principle—the use of an inert viral immunogen to stimulate a primarily humoral immune response in vaccinees—is the same for all IIVs [1]. Indeed, the investigational influenza vaccines of the 1940s were produced in a markedly similar fashion to the egg-grown IIVs of today [2], and subsequent improvements in influenza vaccines (Figure 1) have been incremental rather than revolutionary.

Until recently, vaccine development was an empirical, trial-and-error process. Strategies that worked against one infectious disease were tried against another, with varying levels of success [3]. The infectious diseases against which we still lack effective vaccines are those for which the old strategies have failed. Thus, in recent decades, vaccine development has shifted toward rational design, in which scientific knowledge about the specific pathogen–host interaction guides the vaccine development process [4]. For influenza vaccines specifically, pre-clinical and clinical studies have focused on new delivery platforms—virus-like particles [5], viral vectors [6], nucleic acids [7,8], peptide epitopes [8], nanoparticles [8]—and on novel immunogens that mimic evolutionarily conserved, functionally constrained parts of the virus [5,9,10] or that enhance the recruitment of cell-mediated [10,11,12] or mucosal [6,13] immune responses. These new approaches may significantly improve the effectiveness of influenza vaccines of the future.

Rather than focusing on these novel technologies and immunogens themselves, this review will examine the problem that they are trying to solve—the significant morbidity and mortality attributable to influenza disease in humans—from a primarily clinical perspective. It will explore the clinical outcomes that next-generation influenza vaccines could aim to achieve, and highlight the specific challenges that influenza viruses present for future vaccines to overcome. In particular, it will draw illustrative parallels with other vaccines that provide meaningful clinical benefits, even in the absence of complete sterilizing immunity, which can and should inform the development of future influenza vaccines. Finally, it will identify knowledge gaps in our medical evidence base about influenza disease, which future clinical research could help to close.

## 2. A Vaccine That Prevents Infection

The gold standard for vaccines against infectious diseases is sterilizing immunity—complete protection from infection, which remains durable over years or a lifetime. Many of the earliest and most successful vaccines were developed to target the once-common acute infections of youth, such as measles, mumps, rubella, and smallpox. Even in ancient times, it was known that for certain diseases, people who survived the initial infection developed long-lasting immunity against reinfection [26]. These examples of naturally acquired sterilizing immunity served as proof of principle that immunization was conceptually feasible, if the immunogen could stimulate the same immune response as the pathogen itself [27].

These early vaccines not only conferred long-lasting immunity within a vaccinated individual, but they themselves have also been remarkably durable over time. The live attenuated virus vaccines against measles, mumps, and rubella were developed in 1968, 1967, and 1979, respectively; the same vaccines remain in use today, now administered in combination as the MMR vaccine [28]. Even earlier than that, in 1796 Edward Jenner inoculated young James Phipps against smallpox, using purulent material taken from infectious “pocks” on the hands of a dairy milker, Sarah Nelmes. Because of Nelmes’ occupation, the virus that infected her hand and then protected Phipps from smallpox was assumed to be a cowpox virus, but its true identity remains a mystery. Jenner called his inoculant “vaccinia” (from the Latin for “cow”) even when he obtained it from infected horses [29,30], and the vaccinia virus in all modern smallpox vaccines is most genetically similar to horsepox viruses [29,30,31]. Still, Jenner’s 1796 vaccine was identical in concept, if not in species of origin, to the smallpox vaccine with which the global campaign to eradicate smallpox was won centuries later. MMR and vaccinia serve as examples of the relative simplicity of achieving durable immunity against “one and done” infectious diseases like measles and smallpox.

For infectious diseases in which a single exposure does not confer long-term immunity from reinfection, it is far from trivial to design a human-made vaccine to accomplish what natural infection itself cannot. Some pathogens that cause chronic infections, like *Treponema pallidum* (the causative agent of syphilis) and hepatitis B virus (HBV), are adapted for stealth, highly evolved to fly under the human immune radar for years. Others, like human immunodeficiency virus (HIV) and hepatitis C virus (HCV), take a less silent approach, instead actively interfering with immune response mechanisms on many levels [32,33,34,35]. For these diseases, vaccines are being called upon to stimulate an immune response against pathogens that are exquisitely well adapted to avoid immune-mediated destruction.

Other pathogenic bacteria and viruses can overwhelm with diversity, existing in multiple antigenically distinct serotypes that cause human disease of varying severity but do not induce cross-protective immune responses (e.g., *Streptococcus pneumoniae* [36], *Haemophilus influenzae* [37], human papilloma virus [38], enterovirus [39], and dengue virus [40]). Vaccines against these kinds of pathogens must generally choose a few of perhaps dozens of serotypes to target, usually the most common ones or those that cause the most serious disease. The three genera or *types* of influenza virus that infect humans—influenza A, B, and C—are antigenically distinct from each other, such that infection with one type does not confer immunity to another. The influenza A and B viruses are further subdivided into antigenic clusters called *subtypes* (for influenza A) or *lineages* (for influenza B). While the terminology is different, influenza virus types, subtypes, and lineages are essentially unique serotypes, four of which are currently endemic in and cause clinically significant disease in humans (influenza A/H1N1, A/H3N2, B/Yamagata, and B/Victoria).

Adding to this complexity, each of these unique serotypes is constantly undergoing an evolutionary process called *antigenic drift*. Antibodies recognizing proteins expressed on the surface of influenza virus particles can neutralize virus infectivity, particularly those targeting the receptor-binding protein hemagglutinin (HA). However, the most outward-facing portion of the HA protein is both highly immunogenic and highly tolerant to structural changes at the amino acid level [41]. Random amino acid mutations introduced into this highly plastic region of the HA do not affect the function of the HA protein but can interfere with antibody recognition, so influenza viruses circulating among previously infected humans are under selective pressure to evolve structurally insignificant amino acid changes in the most immunogenic sites. Antigenic drift is the accumulation of these relatively minor changes over time, resulting in influenza virus variants that are no longer effectively neutralized by antibodies made in response to a prior infection. This process renders the human host susceptible to reinfection by the drifted strain [42]. It is this antigenic plasticity that makes influenza virus a unique problem for vaccine development, even in comparison to pathogens that have high serotypic diversity but remain otherwise antigenically stable over many years.

Furthermore, numerous HA serotypes are found in influenza A viruses that are enzootic in other non-human species—wild waterfowl and shore birds, swine, horses, dogs, and bats—to which humans are largely immunologically naïve. Although these HA proteins are most well adapted to infect the species in which they are found, occasionally a human influenza virus can acquire the HA gene from an animal virus. If that HA proves to be good enough at binding to human respiratory tract cells, the resulting virus may be able to begin circulating in the human population, which will have no preexisting immunity to it. This process, called *antigenic shift*, enables pandemic influenza by generating an influenza virus to which humans are susceptible but immunologically naïve [42]. Animal influenza viruses serve as a great pool of antigenic diversity, into which we know that human viruses periodically dip. However, we still do not fully understand all of the critical viral characteristics that confer pandemic potential on non-human influenza A viruses [43].

Despite these influenza virus-specific complexities, complete sterilizing immunity remains the goal for influenza vaccines. Large randomized controlled clinical trials of influenza vaccine efficacy most often use prevention of laboratory-confirmed influenza virus infection as the primary endpoint [44,45,46,47,48], as do vaccine effectiveness estimates by the Centers for Disease Control and Prevention (CDC) in real-world observational cohorts [49]. However, in all of these settings, laboratory testing for influenza virus is generally triggered by the presence of symptoms of an influenza-like illness, almost certainly missing asymptomatic or minimally symptomatic infections that do not prompt a visit to a physician’s office or clinical trial site for evaluation. Even so, the poor effectiveness of influenza vaccines (ranging between 10% and 60% since 2005 [49]) is due in large part to our desire to achieve sterilizing immunity as the benchmark for effectiveness. We are, in essence, asking our influenza vaccines to stimulate a higher level of protection from our immune systems than even natural influenza virus infection can produce.

## 3. A Vaccine That Ameliorates Influenza Disease

Although sterilizing immunity—preventing infection entirely—is a desirable goal for influenza vaccines, not every vaccine can or even must achieve it to confer meaningful clinical benefit. Currently licensed pneumococcal vaccines confer only modest protection against *Streptococcus pneumoniae* pneumonia in adults. However, the rarer and more fatal complication of invasive pneumococcal disease (IPD) is reduced by approximately three-quarters in vaccinated elderly adults [50,51,52]. Bacille Calmette-Guérin (BCG), an attenuated strain of *Mycobacterium bovis*, has been administered to nearly 4 billion people worldwide since 1921, primarily to young children in whom it is highly effective at preventing the potentially fatal complication of disseminated tuberculosis. However, BCG vaccination does not prevent acquisition of latent tuberculosis infection (LTBI), its protection is not long-lasting, and it is relatively ineffective when given to adults, particularly in preventing the reactivation of LTBI into active pulmonary tuberculosis [53]. However, the drastic reduction in pediatric mortality that BCG provides makes the vaccine both clinically effective and cost effective in parts of the world where *M. tuberculosis* remains endemic [54].

Recent studies have reached conflicting conclusions about the ability of current influenza vaccines to modify disease severity among vaccinees who become infected with influenza virus [55,56,57]; these differences may depend on the influenza season under study, and the relative effectiveness of that year’s influenza vaccine against circulating strains. Certainly, though, far more clinical trials have been performed in which sterilizing immunity is the primary outcome under investigation. Far fewer studies have looked at the amelioration of disease as an endpoint, and thus we know far less about whether influenza vaccines function to prevent morbidity and, if so, what viral, host, or seasonal factors contribute year to year to better clinical outcomes.

More research is needed to understand the basic host and viral factors that impact the severity of influenza disease in humans. Morbidity in influenza is largely a consequence of the human immune response to the invading virus. Because influenza viruses are intracellular pathogens, the innate and adaptive immune systems mobilize cytotoxic effector mechanisms to kill infected host cells and in so doing contain the replicating virus. While cytotoxic responses are necessary to check the infection, they sometimes inflict host cell damage that is disproportionate to the viral insult [58]. It is generally accepted that immune dysregulation contributes to severe influenza, but our understanding of the underlying mechanisms, and particularly the genetic or acquired factors that may predispose towards harmful immune responses, remains incomplete [59,60,61,62,63]. An optimal vaccination platform would stimulate protective immune responses to influenza viruses and steer away from those that may contribute to influenza morbidity, particularly in persons with underlying heritable or non-heritable susceptibility to severe disease. However, until we understand more about the clinical characteristics and laboratory markers that are associated with influenza disease severity, it will be difficult to rationally design and study novel vaccines with that goal in mind.

## 4. A Vaccine That Prevents Influenza Virus Transmission

From a public health perspective, an optimal influenza vaccine would both prevent morbidity and mortality in individuals and also inhibit transmission in a population, which currently licensed IIVs cannot reliably achieve [64,65].

The indirect benefits of vaccination have been documented many times in epidemiological studies. After the introduction of the heptavalent pneumococcal conjugate vaccine (PCV7) in children, a gradual but significant decline in the incidence pneumococcal disease in elderly adults was observed, mainly in disease caused by the seven serotypes of pathogenic *S. pneumoniae* included in the vaccine. Reduced colonization with these seven serotypes in vaccinated children likely indirectly benefitted their grandparents’ generation by eliminating these children as a source for *S. pneumoniae* transmission [36,66]. In Japan, a governmental program to vaccinate school-aged children against influenza, in place from 1962 to 1994, was associated with a dramatic decline in excess deaths attributed to pneumonia and influenza, mainly in elderly adults. Excess mortality rates began climbing again when the law requiring vaccination of school children, enacted in 1977, was repealed in 1987. This effect was unlikely due to direct immunization of adults; influenza vaccine coverage in Japanese adults during this period was much lower than in school-aged children, and Japanese health authorities did not issue an official recommendation for influenza vaccination of the elderly until 1997 [67]. The data, both for pneumococcus and influenza virus, suggest that reducing the pathogen burden in children through vaccination can confer clinical benefit in less immunologically robust seniors.

However, despite nearly a century of influenza research, we still have an inadequate understanding of the critical host, viral, and environmental factors that not only determine the efficiency with which influenza viruses transmit from one person to another, but also that govern global influenza virus circulation patterns. Truly basic questions—such as whether influenza viruses are spread primarily by contact with contaminated surfaces or by inhaling airborne viral particles—remain unanswered [68]. 

Critically, we have an incomplete understanding of the viral determinants of efficient respiratory transmission of influenza viruses; these may not be the same parts of the virus that are targeted with vaccines to induce sterilizing immunity. The viral neuraminidase (NA) is a surface-expressed protein that promotes efficient virus release from infected cells by enzymatically cleaving off cell-surface receptors. In animal models, the efficiency of influenza virus transmission correlates with the amount of infectious virus particles actually released into the air, more so than with peak virus titers in the animals’ nasal lavage samples [69]. The enzymatic activity of NA in promoting efficient virus release may explain prior observations that NA function is a critical determinant of efficient mammalian transmissibility of influenza A viruses [70,71,72]. However, current vaccines do not exploit this potential weakness in the transmission chain, and vaccines purposely designed to thwart NA enzymatic activity are in their infancy [73].

Similarly, human immune responses that either promote or inhibit influenza virus transmission are poorly understood. IIVs primarily elicit an antibody response against the immunodominant HA protein. These antibodies prevent influenza virus from engaging with and thus infecting target cells within the human respiratory tract, and the amount of antibodies that a person produces in response to influenza vaccination or viral infection is easily measured in blood samples by the in vitro hemagglutination inhibition (HI) assay. Anti-HA antibody production in response to a candidate influenza vaccine, as measured by the HI assay, is an accepted “correlate of protection” [74]—a laboratory test result that is predictive of a vaccine’s ability to prevent infection or disease. In other words, the lab test result correlates with the clinical efficacy of the vaccine in a population that receives it. However, we have an incomplete understanding of the correlates of protection that apply in particular to influenza virus transmissibility.

Vaccination in general [75] and mucosal anti-HA immunity specifically [76] have previously been shown to block influenza A virus transmission in the guinea pig model. Immunizing guinea pigs with a chimeric mouse/guinea pig monoclonal IgG against the influenza A HA, and then infecting the immunized animals with the homologous virus, could delay but not prevent subsequent virus transmission to naïve partner animals, even though high serum HI titers were achieved in vaccinated guinea pigs. However, immunizing guinea pigs with an isotype-switched IgA version of the same chimeric anti-HA antibody could delay or even abolish transmission in a dose-dependent manner, and the IgA antibody was detectible by enzyme-linked immunosorbent assay (ELISA) only in the nasal washes of guinea pigs immunized with a dose sufficient to block transmission [76]. Together with animal studies demonstrating that a live-attenuated influenza vaccine (LAIV) confers greater protection from heterologous influenza virus challenge than IIV [75,77] and human data showing that secretory IgA (sIgA) responses are significantly boosted in adults receiving LAIV but not IIV [78], these observations suggest that influenza virus transmission might be inhibited by vaccines improved to optimize mucosal immune responses, which are poorly induced by IIVs. A more complete understanding of the viral and host factors that drive or impede influenza virus transmission, as well as associated laboratory correlates of protection on both individual and population levels, will be critical in developing improved influenza vaccines.

## 5. A Vaccine That Benefits Everyone

Seasonal influenza tends to be most severe at the extremes of age—at one end, infants and very young children, and at the other end, the elderly. Unsurprisingly, these are also the age groups that mount the weakest antibody responses to IIVs, though they are in the greatest need of protection. Though anti-HA antibody production, as measured by the HI assay, is an accepted correlate of protection for IIVs [74], it is important to note that for an individual person, post-vaccination HI titer does not always correlate with protection from infection; vaccine failures do occur even in those with robust HI titers [79]. Also, the established HI-based correlates of protection may not be the best predictors of IIV efficacy in some groups at risk for complicated influenza, like children [80], the elderly [81], or obese persons [82]. In other high-risk groups, such as persons with underlying respiratory [83,84] or kidney [85] disease, and pregnant women and their newborn infants [86], IIV effectiveness data are relatively scarce overall.

LAIV activates the immune system differently and does not stimulate HI antibody titers to the same degree as IIVs, especially in children [87,88]. However, its efficacy in preventing influenza in children, particularly in the pre-school age group, is generally equal or superior to IIV [89,90,91,92]. Like LAIV, the clinical efficacy of many future influenza vaccines—such as those targeting antibody epitopes other than the HA receptor-binding domain, and those specifically designed to stimulate mucosal or cell-mediated immune memory—may not correlate with the serum IgG responses, as measured by the HI assay, in any age group.

**Figure 2:**
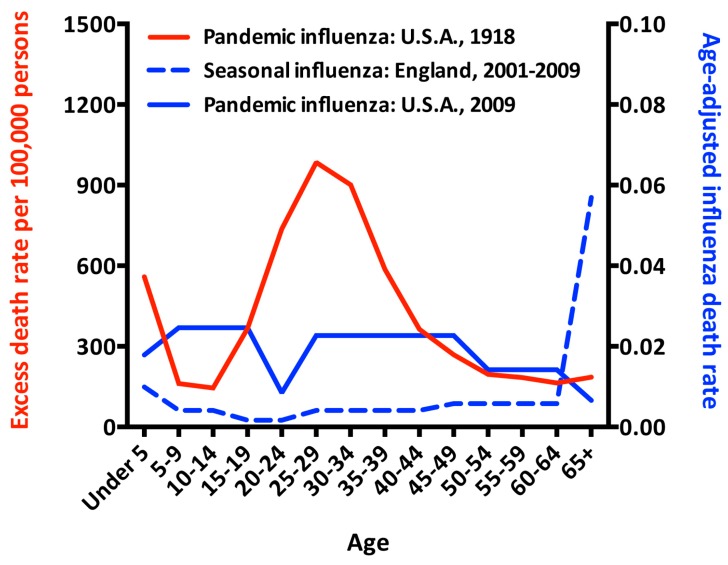
Influenza deaths in pandemic and epidemic influenza. Data from the pandemic year of 1918 are graphed against the left y-axis as the excess death rate above the baseline death rate observed in the non-pandemic year of 1914 [93]. Data from 2001–2009 are graphed against the right y-axis as the age-adjusted death rate (AADR), calculated as follows: Influenza-attributed crude death rate per 100,000 population (CDR) = the number of influenza deaths in each age group ÷ the population in that age group × 100,000; AADR = CDR × the proportion of the entire population within that age group. Influenza death counts by age group were obtained from published data for the U.S. [95] and England [94]. U.S. census figures from 2010 [96] and U.K. national population estimates for England (code E92000001) in mid-year 2005 [97] were used to approximate the number and age distribution of the population at risk during each time period.

Age-related changes in immune system function may explain why the very young and the very old do not mount similar immune responses to influenza vaccines as non-elderly adults. However, age may also be a surrogate marker for immune history, the accumulated memory of a lifetime of exposure to influenza viruses or vaccines. In pandemic influenza, the usual severity pattern—often described as a “U”-shaped curve, with morbidity and mortality concentrated at the extremes of age—takes on different forms, with younger people typically suffering disproportionately severe disease compared to seasonal epidemic influenza (Figure 2). Although overall annual death rates were higher in every age group in the pandemic year of 1918, compared to the non-pandemic year of 1914, the excess death rate was proportionally far larger in young and middle-aged people than in the elderly [93]. Similarly, in non-pandemic years, the elderly die of influenza at much higher rates than any other age group [94], but in the second wave of the 2009 pandemic (August to October, 2009), that trend was reversed, and the elderly had the lowest influenza-attributable death rate of any age group [95]. 

Like pandemic influenza, zoonotic influenza is typically caused by an HA serotype to which the infected person is immunologically naive. In humans, two avian influenza A subtypes, H5N1 and H7N9, seem to cause the most severe disease in different age groups. For H5N1 infections reported to the World Health Organization (WHO), the median age of cases is 18 years, with a case fatality rate of 60%, while the median age of H7N9 cases is 58 years, with a case fatality rate of 40% [98]. Epidemiological explanations have been offered to explain this age discrepancy, but there is also scientific evidence that the types or subtypes of influenza viruses to which humans were exposed very early in life can affect subsequent immune responses to antigenically novel viruses [99], which are characteristic of both zoonotic and pandemic influenza.

Through antigenic drift and shift, the influenza viruses circulating throughout the human population are constantly changing over time. Thus, different birth cohorts experience first infections with different predominant strains, and we are beginning to understand how these varied early exposures leave different “immune imprints” in these cohorts. How similar or different a never-before-seen influenza virus is to the strain with which an individual was “imprinted” in early childhood may influence the potency with which his or her immune system can respond to it [100,101,102]. If this hypothesis is correct, certain people will be more susceptible to severe disease from novel pandemic or zoonotic strains simply based on when they were born, regardless of other high-risk factors they may or may not possess. Influenza vaccines that confer a greater breadth of protection against heterologous or heterosubtypic influenza virus strains are certainly desirable, but the strategy for achieving greater breadth may have to account for differences in age-related immune imprints to be equally beneficial to people across the age spectrum.

## 6. A Vaccine That is Accepted

No clinical discussion about improving influenza vaccines is complete without acknowledging that current influenza vaccines’ lack of effectiveness on a population level is due in part to poor uptake by those eligible to receive it. In the United States, influenza vaccine coverage remains poor—ranging from 70% among the elderly to 32% of young and middle-aged adults [103]—despite a near-blanket recommendation for vaccination of all persons above 6 months of age in the absence of contraindications, of which there are very few [1].

Vaccine hesitancy is not unique to influenza vaccines, nor is it due entirely to unfavorable opinions about their suboptimal effectiveness and durability. Other vaccines that do not induce complete sterilizing immunity but still confer clinical benefit, such as pneumococcal vaccines and BCG, are widely accepted [104,105]. In contrast, the HPV vaccine faces acceptance hurdles [106,107], despite clear and compelling scientific evidence that it safely and effectively prevents cancer in those who receive it in a timely fashion [108]. Vaccine hesitancy, particularly for those vaccines given in the adolescent and non-elderly adult years, clearly has a psychosocial component that we are only beginning, through research, to learn how to address [106,107,109,110,111,112,113].

## 7. Conclusions

New influenza vaccine strategies and platforms are coming [114,115,116], based upon a better understanding of influenza viruses and human host responses to them. Although durable sterilizing immunity is the ultimate goal, real clinical benefit would be gained from vaccines that reduce the transmissibility of influenza viruses or the severity of disease that they cause, or that increase the breadth of protection against heterologous influenza virus serotypes that humans may encounter. In the absence of sterilizing immunity, we should not consider improvements in these alternative outcomes as failures.

Still, it remains to be seen in clinical trials whether these rationally designed approaches, either alone or in combination, will offer superior protection from influenza morbidity or mortality in humans—and importantly, if and how influenza viruses might evolve to find ways to get around them. With “tricky” pathogens like influenza viruses, we must maintain a degree of tolerance for unintended consequences.

After the introduction of the PCV7 pneumococcal vaccine, which covered the seven *S. pneumoniae* serotypes causing the majority of invasive pneumococcal disease (IPD) in children, the incidence of IPD dropped dramatically across all age groups. When IPD did occur, though, it was caused largely by non-vaccine serotypes, which have since been included in the expanded 13-valent PCV13 vaccine. Since PCV13, however, new data from England and Wales suggests that IPD with non-PCV13 serotypes—in particular, serotypes 8, 12F, and 9N—has been rising faster than disease caused by the additional six serotypes in PCV13 has been falling, essentially offsetting the gains offered by PCV13 [117]. While these data await confirmation elsewhere, they are a reminder that many infectious pathogens have the evolutionary capacity to confound human efforts to contain them, in ways that cannot always be foreseen.

As much as we have learned about influenza viruses in the past century, much about this particular host–pathogen interaction remains unknown. New vaccine technologies are being discovered at a rapid pace; however, our sense of how to implement these technologies to optimal clinical benefit lags behind. Future medical research is needed to better understand immune responses that correlate with ameliorating disease and preventing population-level transmission, particularly in high-risk groups like the very young and the very old, pregnant women, and people with comorbidities known to worsen influenza outcomes. With a pathogen as complex as influenza virus, however, we are unlikely to know everything that we need to know to design the perfect vaccine. While rationally designed improvements in vaccine technology hold great promise for the future, an element of trial and error inevitably remains as we try to combat the problem of influenza.
